# Eight quick tips for biologically and medically informed machine learning

**DOI:** 10.1371/journal.pcbi.1012711

**Published:** 2025-01-09

**Authors:** Luca Oneto, Davide Chicco

**Affiliations:** 1 Dipartimento di Informatica Bioingegneria Robotica e Ingegneria dei Sistemi, Università di Genova, Genoa, Italy; 2 Dipartimento di Informatica Sistemistica e Comunicazione, Università di Milano-Bicocca, Milan, Italy; 3 Institute of Health Policy Management and Evaluation, University of Toronto, Toronto, Ontario, Canada; SIB Swiss Institute of Bioinformatics, SWITZERLAND

## Abstract

Machine learning has become a powerful tool for computational analysis in the biomedical sciences, with its effectiveness significantly enhanced by integrating domain-specific knowledge. This integration has give rise to informed machine learning, in contrast to studies that lack domain knowledge and treat all variables equally (uninformed machine learning). While the application of informed machine learning to bioinformatics and health informatics datasets has become more seamless, the likelihood of errors has also increased. To address this drawback, we present eight guidelines outlining best practices for employing informed machine learning methods in biomedical sciences. These quick tips offer recommendations on various aspects of informed machine learning analysis, aiming to assist researchers in generating more robust, explainable, and dependable results. Even if we originally crafted these eight simple suggestions for novices, we believe they are deemed relevant for expert computational researchers as well.

## Introduction

Machine learning has become pervasive in a huge number of computational biology and medicine studies nowadays to address complicated problems being the backbone of the most novel research [1]. In fact, computational intelligence models help scientists understand complex biological processes [2], predict outcomes of a medical procedure [3,4], and support the design of new drugs [5]. Nevertheless, mistakes and bad practices in applying computational intelligence to biomedical data have become common, too [6–8].

The machine learning approaches can be nowadays categorized into two groups: informed and uninformed [9,10]. We call uninformed machine learning the models that do not make prior assumptions about the data set, meaning that they treat all the variables and the instances of the dataset in the same way, egalitarianly. These models do not take into account the biomedical knowledge that would beforehand highlight the role of a particular set of factors. On the contrary, we call informed machine learning the models which do take into account knowledge about the data set scientific subfield, during data collection and preparation (data pre-processing), during model development (in-processing), or during model correction and alignment (post-processing).

A study where a computational feature ranking phase is done on data of electronic medical records by treating all the variables in the same way [11], for example, can be considered an uninformed machine learning project. On the other hand, a bioinformatics study where three particular genes are known to be related to neuroblastoma and therefore treated with more importance compared to other genes [12] can be called informed machine learning.

It should be noted that the concepts of informed machine learning and uninformed machine learning have been previously introduced and developed in the field of artificial intelligence. These terms have their origins in background learning, which was first introduced in the 1980s. It is noteworthy that Art Samuel [13] was among the first to combine learning and domain knowledge-defined structure of the model, which could be considered the earliest example of informed machine learning, in 1959. Currently, there is a wealth of literature in this field that also proposes a historic perspective [14,15].

Before diving into the tips, let us first clarify what informed computational intelligence models are, when to use them, and help you recognize that you are likely already using them in many situations.

In order to understand what are the biologically and medically informed (partial-knowledge) computational intelligence models [16–18] and how to use them we have to start from the two most established approaches: the knowledge-based [10,16,19] (full-knowledge) and the data-driven based [11,20] (zero-knowledge) models.

Full-knowledge models strongly rely on humans and their comprehensive domain knowledge, employing a relatively small subset of available data and simple statistics primarily for validation rather than for model construction [10,19]. They often do not fully exploit all the possibly available data since some of them may be hard to exploit just based on domain knowledge [21]. Full-knowledge models are characterized by their predictability (as they are explainable by design) and adherence to physical plausibility, making them particularly suitable for biological and medical applications where the underlying mechanisms are well understood [22,23]. However, the effectiveness of these models is limited by the human capacity to conceptualize and manage complex biological systems, rendering them less flexible for novel or poorly understood phenomena [24–26].

Zero-knowledge models utilize large datasets to build and validate models without prior domain knowledge [27–30]. These models harness the computational power of modern technological infrastructures to analyze data, to identify patterns, and to make predictions that may not be immediately apparent to human researchers [31–34]. While these models excel in handling vast amounts of data and making generalized predictions, their outputs may not always be aligned with physical or biological plausibility or understandable and explainable, especially when extrapolating beyond the scope of the data; this limitation underscores the necessity for cautious interpretation of results, particularly in biological and medical settings where point wise accuracy and understanding the underlying mechanisms are crucial [35–39].

Partial-knowledge models represent a synthesis between the full- and zero-knowledge models capitalizing on the predictability, explainability, and plausibility of knowledge-based models while leveraging the data-processing capabilities of data-driven models. By integrating domain knowledge at varying levels, from data collection and feature engineering to the inclusion of approximate system models and model post-processing, partial-knowledge models strive for accuracy in both general and specific instances, enhancing their utility in biological and medical research for both interpolation and extrapolation tasks [10,16–19].

The integration of these modeling approaches within the biological and medical sciences can potentially enhance our understanding and treatment of complex diseases [40], facilitate the discovery of new therapeutics [41], and contribute to personalized medicine [42]. By properly selecting and combining these methodologies, researchers can navigate the intricacies of biological systems and medical conditions, advancing the frontier of healthcare innovation [10,16–19].

Note that, some of the readers might have never heard about the terms informed machine learning and uninformed machine learning, and might read this manuscript thinking they are discovering something new. However, it is actually likely that they have already completed computational projects in both these areas in the past already.

The biomedical literature contains plenty of articles involving data-driven feature synthesis and selection, which could be categorized on informed machine learning (for example, [12,14]).

**Fig 1:**
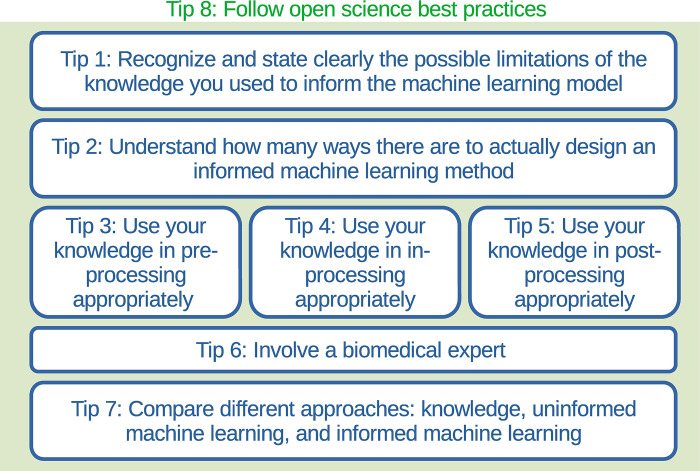
A schematic representation of the flowchart of the execution of our eight guidelines. The best practices for open science should be followed from the beginning to the end, and therefore, we represented them as the background of the whole process.

Both informed and uninformed machine learning have advantages and drawbacks and are error-prone. The Quick Tips series published articles on several computational aspects in the past, but none on this topic. We fill this gap by providing our eight quick tips for avoiding common mistakes and pitfalls when using informed machine learning in the biomedical sciences (Fig 1). We originally designed our recommendations for novices, but we believe they should be kept in mind by experts, too.

## Tip 1: Recognize and state clearly the possible limitations of the knowledge you used to inform the machine learning model

The usage of informed machine learning, as explained earlier, can bring several advantages to a scientific study. But it can, however, generate some problems as well.

Therefore, it is always important to keep in mind the limitations of this approach [43].

The informed machine learning approach, in fact, does not allow an agnostic inclusion of all the biomedical variables into a statistical model, and thus this selection sometimes can result being misleading [15]. For example, if the knowledge introduced by the informed model was wrong, outdated, obsolete, or misleading, it would corrupt the statistical model and generate inaccurate or inflated outcomes and results eventually. Sometimes, the information might be available as a general knowledge, but no specific feature related to that knowledge might have been annotated in the data set.

Occasionally, the biomedical experts and the computer scientists, although willing to collaborate, might not understand each other because of different experiences, knowledge, and jargon [44].

Other setbacks might happen because of the complexity of the model: including knowledge might seem useful, but if the statistical model became too complicated to be handled correctly, of course there would be no final benefit for the study.

Moreover, an informed machine learning model might fail to assimilate the knowledge introduced, and therefore might still work agnostically, even if the person who prepared the model thought they were preparing an informed machine learning algorithm [15,43].

Finally, past research has shown that adding additional knowledge to model inference might either increase or decrease the accuracy of the resulting models [45,46].

## Tip 2: Understand how many ways there are to actually design an informed machine learning method

As described earlier, biologically and medically informed machine learning is an innovative approach that integrates domain-specific knowledge into data-driven models enhancing their performance, explainability, and plausibility [10,16,19]. This integration can be implemented at various stages of the machine learning pipeline, primarily categorized into pre-, in-, and post-processing methods.

Pre-processing is a critical step that involves preparing and transforming the data before it is fed into a machine learning model [47,48]. This stage is pivotal because it directly addresses the quality of input data, ensuring that the machine learning model has the best possible starting point. Techniques such as data cleaning [49], feature engineering [50,51], and data augmentation [52] fall under this category, where domain knowledge is leveraged to enhance the data set’s relevance and quality. Furthermore, full-knowledge models that utilize domain knowledge to provide a first hint to be fed to or to be corrected by a machine learning model (that means, commonly referred as serial or parallel informed models) exemplify how domain knowledge can guide the machine learning model towards more accurate and relevant predictions [10]. Pre-processing can also improve explainability as machine learning model trained on a richer set of features and higher quality data can lead to simpler and then explainable models [17,53,54].

Pre-processing essentially capitalizes on domain expertise to navigate the machine learning model through the complex data landscape, minimizing the distance it needs to cover to generate valuable insights [55]. The pre-processing approach has been employed in several biomedical informatics studies in the past, especially when specific biomarkers were deemed more important than others before a computational phase [12].

In-processing involves the direct incorporation of domain knowledge into the computational intelligence model’s learning process [56]. This method requires a deep integration of mathematical representations of domain insights (such as laws, trends, or constraints) into the learning algorithm itself [56] and demands a collaborative effort between domain experts and data scientists to modify the learning algorithm’s structure [57]. This necessity could mean altering the functional form of the model [28,30] (for example, a particular architecture of a neural network), introducing specific constraints [58], or embedding regularizers [59] to maintain the model’s desirable properties like convexity [60] and differentiability [61]. The objective is to steer the model’s learning mechanism in a way that it not only benefits from domain knowledge but also enhances its predictive accuracy on a granular level, beyond average performance metrics [62]. By selection the proper model in-processing can also deal with the trade-off between accuracy and explainability [53]. The in-processing approach has been employed in multiple biomedical informatics studies in the past [10,56].

Post-processing focuses on refining the machine learning model’s outputs to ensure they align with domain knowledge and expectations [63,64]. This stage does not modify the machine learning model itself but adjusts its outputs through additional rules or models to enforce domain consistency [65]. This alignment can improve also explainability by, for example, forcing the model to be never too far from a full-knowledge model [17,53]. Techniques include using machine learning predictions as inputs to physical models for more controlled outcomes or applying logical rules to rectify inconsistencies in predictions [66,67] (for example, a prediction indicating cancer should not concurrently suggest healthiness). Post-processing is about leveraging the existing machine learning capabilities as-is and employing domain knowledge to contextualize and correct the model’s predictions [68]. This approach aims to mitigate potential errors and align the model’s outputs with domain-specific truths, requiring substantial domain understanding to implement effectively [69,70]. The biomedical informatics literature has plenty of studies reporting post-processing informed machine learning approaches [10,19].

## Tip 3: Use your knowledge in pre-processing appropriately

Pre-processing plays a crucial role in enhancing the predictive model performance by leveraging domain knowledge to inform, a priori, the machine learning model effectively [9,71].

Pre-processing acts, a priori, in different ways:

- Modifying the input (that is, the features) fed to the machine learning models;
- Modifying the data (that is, the observation or samples) used to train the machine learning models; and
- Guiding the selection of the type of machine learning algorithms.

Modifying the input means cleaning, cleansing, engineering, selecting, and reducing the inputs to remove inconsistencies and errors, and enrich the input to ensure that the information fed into the machine learning models is of high quality [49,72].

Modifying the input also means constructing serial or parallel biologically and medically informed machine learning. In serial and parallel informed machine learning, a potentially partial (meaning that is unable to take into account all the available data) full-knowledge model is available to make prediction [73].

Modifying the data set involves selecting and enriching the available data [74], not only by choosing the most appropriate data but also by designing experiments to collect this data if necessary [75], ensuring it accurately represents the phenomena under study [76]. Techniques for data fusion and data integration are a key in this context [77].

Experimental design for data collection is probably the most important phase of a successful machine learning-based research project or product as it allows to prevent to introduce spurious correlations [78] and to try to match the main hypothesis behind any machine learning algorithm, namely the data well represent the population [79].

Guiding the selection of the type of machine learning algorithm means guiding the selection of the algorithm functional form [30] (for instance, deep or shallow and for the deep the type of architecture), including transfer learning [80], the level of explainability of the model (from rule based model to deep models passing from linear models) [53], and the hyperparameters characterizing the machine learning algorithm [81,82]. This aspect is a pivotal part of the process and should be informed by the specific characteristics of the pre-processed and enriched data set and the physiological mechanisms/principles and by the domain knowledge underpinning the problem space [83].

For example, if we are in a safety-critical situation, it is better to use a fully interpretable model [53], even if less accurate. If we deal with images, convolutions are the best choice, while transformers are the way to go if we have to deal with natural language [28]. If we have a lot of structured data, deep models are probably the best choice, while for medium or small cardinality unstructured data sets, shallow models are the optimal choice [30]. This mix of domain knowledge and experience can make a difference in delivering an effective biomedically informed machine learning study.

## Tip 4: Use your knowledge in in-processing appropriately

In-processing plays a crucial role in enhancing the predictive model performance by leveraging domain knowledge of a potentially partial, but well mathematically encoded, full-knowledge model to inform the learning process of a zero-knowledge effectively [56].

In-processing involves the integration of domain-specific laws and principles directly into the model training process, especially to ensure that the models adhere closely to known scientific knowledge [84]. When certain biological laws or medical principles are known, these can be used to guide the model in several ways [69]. One approach is to ensure the model’s predictions do not deviate significantly from these laws by incorporating a regularization term that penalizes deviations from the expected physical behavior [68]. This regularization can help in maintaining the model’s fidelity to the biological laws or medical principles, such as the relationship between specific inputs and outputs, ensuring that if an input value increases, the output adjusts in a biological or medical consistent manner [68,69]. Moreover, when dealing with complex systems where simulators exist but are too slow for practical use, surrogate models can be developed [84]. These models aim to mimic the simulator’s outputs while being computationally efficient, thus requiring the model to accurately capture the underlying physical relationships [56]. Incorporating biological laws or medical principles into machine learning does not always require exhaustive or precise details; even hints or partial knowledge about the physical system can be beneficial [10,56]. Other methods like the ones based on reasoning like inductive logic programming [85], neuro-symbolic approaches [67,86], and learning constrained models [58] have also shown to achieve good practical results.

In summary, the in-processing strategy within biologically and medically informed machine learning represents a paradigm shift toward developing machine learning models that are informed by and compliant with the biological laws or medical principles. This approach significantly contributes to creating models that are not only predictive but also interpretative and aligned with the real-world phenomena they aim to simulate, thereby bridging the gap between data-driven insights and biological laws or medical principles.

## Tip 5: Use your knowledge in post-processing appropriately

Post-processing leverages domain knowledge (for example, a potentially, partial but still well-mathematically encoded, full-knowledge model) to align the output of a zero-knowledge model [63].

Post-processing in physics-informed models focuses on refining predictions of a zero-knowledge model to ensure they align with domain-specific knowledge, constraints, and practical considerations [64]. This step is crucial for enforcing certain characteristics and relationships that must be present in the predictions. For instance, in an autonomous system to diagnose different types of cancers, if a model predicts the type of cancer, it must recognize hard constraints such as the hierarchies present in the diseases, namely, if we predict cancer in the lung we also have to predict lung problems [66,67].

Furthermore, post-processing involves adjusting predictions to ensure they do not diverge excessively from established practices, like the dose of chemotherapy, thereby ensuring that the model’s recommendations are practical and implementable within current protocols. This step not only enhances the model’s reliability but also its acceptance among practitioners [63]. Ensuring that the model’s decisions are in harmony with domain knowledge can be straightforward if the model is inherently explainable [53]. For models that are not easily interpretable, techniques such as feature importance analysis can provide global explainability, offering insights into what the model considers important across all decisions [53]. For local explainability—understanding individual predictions—tools can be employed to break down the decision-making process for specific cases [53].

Eventually, one can decide to build full-knowledge model, then interpretable and controllable, that require some inputs that are not easy to measure or estimate: in this case zero-knowledge model might be of support and more easily controllable, since their estimate can be verified and controlled by the subsequent full-knowledge model toward final biological and medical informed machine learning model [64].

## Tip 6: Involve a biomedical expert

As explained in other Quick Tips articles [87], the success of an interdisciplinary scientific project relies massively on the involvement of domain experts for all the scientific fields involved. So, if your study is on bioinformatics, we suggest you to involve a wet-lab biologist, and if your study is on medical informatics, we advise you contact a medical doctor. These biomedical experts need to be contacted and included in two main phases: at the beginning of your project (when you are defining the scientific question) and at the end (when the results are ready and their implications need to be discerned) [88].

Therefore, if you work at a university, we recommend that you contact a biologist in the biology department or a medical doctor in the medicine department [44]. If there are no biomedical experts in your organization, we recommend that you look for someone online, on forums such as Reddit, StackExchange, or similar.

The information given by the biomedical expert will be pivotal: they will provide insights for the scientific question definition and for the result understanding that will be invaluable and that will enrich your study.

Of course, it would be even better if you could have the support of a biomedical researcher throughout the whole project and not just at the start and at the end. This hope, however, can be too optimistic knowing the busy schedule of medical doctors and wet-lab biologists [44].

## Tip 7: Evaluate and compare different approaches: Knowledge, uninformed machine learning, and informed machine learning

One of the best practices of computational projects is to use different methods to see if similar results are found. The same approach can be employed in a biomedical study: therefore, when you have a biomedical data set ready to be analyzed, we suggest you to process it through a knowledge approach (using common knowledge about the scientific subfield and standard statistics about the data set, without machine learning [19]), a uninformed machine learning approach (also called data-driven or zero-knowledge [11,20]), and an informed machine learning approach (also called partial-knowledge [19]).

A knowledge strategy that includes only information about the scientific domain and involves no computational intelligence can be carried out through traditional statistics [89], by checking against common knowledge: for example, if a specific clinical factor is known to be prognostic for the disease of the data set, one can double-check if the results obtained through the informed or uninformed machine learning approaches confirm it or not.

For instance, in a previous study on a data set of electronic health records with chronic kidney disease, we utilized a uninformed machine learning approached which detected age, estimated glomerular filtration rate (eGFR), and creatinine as most diagnostic factors [90]. Afterwards, we double-checked our discoveries in the scientific literature (knowledge approach) to see if they could be confirmed or not. That is what we suggest you to do.

The results of informed machine learning studies [12] should be checked against common knowledge on the disease investigated, too. Comparison of results can lead to interesting insights about the data (for example, if more data might be needed to complete the study), or about the methods (for example, if the results contradict common knowledge), or about new discoveries.

Regarding evaluation, past research has shown that adding additional knowledge to model inference might either increase or decrease the accuracy of the resulting models, but it consistently aids in generating explainable models [45,46].

## Tip 8: Follow open science best practices

Even for informed machine learning, we promote the usage of best practices for open science: open source software code, open data release, and open access publication [91].

If you have the chance to decide which programming language to use for your informed machine learning project, we strongly suggest to pick an open source one, such as Python or R. This way, you will be able to share your software code with any collaborator at any time and, if you publish your software code openly later on GitHub or GitLab, anyone will be able to use it. These practices would ensure the possibility to reproduce and replicate your computational results, and would allow other researchers around the world to start new, similar scientific projects if they want.

Regarding data, we recommend that you share your data online in public, open repositories, if you are authorized to do so. There are several open repositories for bioinformatics data and medical data where you can release your data set (Gene Expression Omnibus (GEO), ArrayExpress, Sequence Read Archive (SRA), and the Cancer Genome Atlas (TCGA), the Cancer Imaging Archive (TCIA), and PhysioNet, for example) and for any data type (Figshare, Zenodo, and University of California Irvine Machine Learning Repository).

We advise you to openly publish both the raw data and the preprocessed data you used for your analysis. Of course, the privacy of data’s patients need to be preserved: make sure that all the data are anonymous, deidentified, and unidentifiable.

Moreover, as explained in other Quick Tips articles [88,92,93], we suggest to use these online resources to look for an alternative data set of the same type and of the same disease of the primary cohort dataset that you analyzed in your study. If you found one, repeat your analysis on it and see if your scientific discoveries are confirmed there.

Finally, if you have a say on which scientific journal to choose for your paper submission, we suggest to pick an open access one: publishing an open access article, in fact, would make it readable and available to anyone in the world, and also let your study have a bigger impact on the scientific community.

## Conclusions

Informed machine learning has become popular in several biomedical studies nowadays, thanks to the large availability of computational resources and the spread of knowledge about computational intelligence. Even if it has become easier to apply informed machine learning, it has become easier to make mistakes, too: bad practices and pitfalls, if not carefully handled, that can produce negative consequences on the final results of the study. In this manuscript, we propose eight simple guidelines for avoiding common mistakes and inaccuracies in studies involving informed machine learning phases.

We believe our eight recommendations can help researchers produce more stable and reliable results in any biomedical study.
